# MiRNA Profiling in Plasma Neural-Derived Small Extracellular Vesicles from Patients with Alzheimer’s Disease

**DOI:** 10.3390/cells9061443

**Published:** 2020-06-10

**Authors:** Maria Serpente, Chiara Fenoglio, Marianna D’Anca, Marina Arcaro, Federica Sorrentino, Caterina Visconte, Andrea Arighi, Giorgio G. Fumagalli, Laura Porretti, Alessandra Cattaneo, Miriam Ciani, Roberta Zanardini, Luisa Benussi, Roberta Ghidoni, Elio Scarpini, Daniela Galimberti

**Affiliations:** 1 Department of Pathophysiology and Transplantation, Dino Ferrari Center, University of Milan, 20122 Milan, Italy; chiara.fenoglio@unimi.it (C.F.); marianna.danca@unimi.it (M.D.); 2 Neurodegenerative Diseases Unit, Fondazione IRCCS Ca’ Granda, Ospedale Maggiore Policlinico, 20122 Milan, Italy; marina.arcaro@policlinico.mi.it (M.A.); federica.sorrentino@unimi.it (F.S.); caterina.visconte@gmail.com (C.V.); andrea.arighi@policlinico.mi.it (A.A.); giorgio.fumagalli@policlinico.mi.it (G.G.F.); elio.scarpini@unimi.it (E.S.); daniela.galimberti@unimi.it (D.G.); 3 Department of Biomedical, Surgical and Dental Sciences, Dino Ferrari Center, CRC Molecular Basis of Neuro-Psycho-Geriatrics Diseases, University of Milan, 20122 Milan, Italy; 4 Flow Cytometry Service, Fondazione IRCCS Ca’ Granda Ospedale Maggiore Policlinico, 20122 Milan, Italy; laura.porretti@policlinico.mi.it; 5 Department of Transfusion Medicine and Haematology, Fondazione IRCCS Ca’ Granda Ospedale Maggiore Policlinico, 20122 Milan, Italy; alessandra.cattaneo@policlinico.mi.it; 6 Molecular Markers Laboratory, IRCCS Istituto Centro San Giovanni di Dio Fatebenefratelli, 25125 Brescia, Italy; mciani@fatebenefratelli.eu (M.C.); rzanardini@fatebenefratelli.eu (R.Z.); lbenussi@fatebenefratelli.eu (L.B.); rghidoni@fatebenefratelli.eu (R.G.)

**Keywords:** Alzheimer’s disease, extracellular vesicles (EVs), neural-derived extracellular vesicles (NDEVs), microRNA

## Abstract

Small extracellular vesicles (EVs) are able to pass from the central nervous system (CNS) into peripheral blood and contain molecule markers of their parental origin. The aim of our study was to isolate and characterize total and neural-derived small EVs (NDEVs) and their micro RNA (miRNA) cargo in Alzheimer’s disease (AD) patients. Small NDEVs were isolated from plasma in a population consisting of 40 AD patients and 40 healthy subjects (CTRLs) using high throughput Advanced TaqMan miRNA OpenArrays^®^, which enables the simultaneous determination of 754 miRNAs. MiR-23a-3p, miR-223-3p, miR-100-3p and miR-190-5p showed a significant dysregulation in small NDEVs from AD patients as compared with controls (1.16 ± 0.49 versus 7.54 ± 2.5, *p* = 0.026; 9.32 ± 2.27 versus 0.66 ± 0.18, *p* <0.0001; 0.069 ± 0.01 versus 0.5 ± 0.1, *p* < 0.0001 and 2.9 ± 1.2 versus 1.93 ± 0.9, *p* < 0.05, respectively). A further validation analysis confirmed that miR-23a-3p, miR-223-3p and miR-190a-5p levels in small NDEVs from AD patients were significantly upregulated as compared with controls (*p* = 0.008; *p* = 0.016; *p* = 0.003, respectively) whereas miR-100-3p levels were significantly downregulated (*p* = 0.008). This is the first study that carries out the comparison between total plasma small EV population and NDEVs, demonstrating the presence of a specific AD NDEV miRNA signature.

## 1. Background 

Alzheimer’s disease (AD) is a chronic neurodegenerative disorder characterized by cognitive decline and memory impairment. The neuropathological hallmarks include the deposition of senile plaques resulting from the excessive accumulation of β-amyloid protein (Aβ) and the formation of neurofibrillary tangles (NFTs) derived from hyperphosphorylated tau proteins leading globally to neural dysfunction [1]. Increasing epidemiological evidence shows that there is a presymptomatic long-lasting period for clinical incubation of the disease that could take several years before leading to cognitive dysfunction. The long preclinical period of AD makes it necessary to seek robust and reliable diagnostic biomarkers for early intervention. At present, diagnostic research criteria for AD [2] include emission tomography (PET) with amyloid tracer, which is a complex and very expensive tool, and the cerebrospinal fluid (CSF) pathogenic protein detection, that implies the invasive procedure of sample collection by lumbar puncture. In this scenario, circulating biomarkers would be of help for differential diagnosis and possibly for monitoring cognitive decline. 

Promising candidates are small extracellular vesicles (EVs) that are extracellular membrane vesicles of endosomal origin with a diameter ranging from 30 to 150 nm. Initially referred to as “exosomes” by the international scientific community, they were later addressed by the International Society for Extracellular Vesicles (ISEV) as “small EVs” [3]. They are produced by all cell types and contain several molecular markers of their parental origin. They also include proteins, lipids and nucleic acids, named EV “cargo”. The EV “cargo” varies depending on the physiological state of the cell [4]. Cells from the central nervous system (CNS), neurons, astrocytes, oligodendrocytes are also able to secrete small EVs into the extracellular compartment, both in physiological and pathological conditions [5]. They could thus reflect the state of their neural cell of origin, an aspect that may contribute to the diagnosis of neurodegenerative diseases [6]. In this regard, Fiandaca et al. were able to isolate small neural-derived extracellular vesicles (NDEVs) from plasma using a combination of chemical and immunochemical approaches based on specific antibody against neural L1 cell adhesion molecule (L1CAM) and to evaluate their protein content [7]. This challenging approach for the isolation of a specific subpopulation of vesicles has generated increasing interest in using small NDEVs as a source for biomarkers in neurodegenerative diseases including AD [8,9,10]. The ability to analyze the content of plasma small NDEVs has been demonstrated on several fronts to be relevant to AD pathogenesis and also highlights the role of small NDEVs as promising biomarkers. This hypothesis has also been supported by increasing evidence including levels of amyloid-*β* and tau proteins, as well as the synaptic proteins [11] involved in AD pathogenesis.

As mentioned above, the EVs’ “cargo” also comprises nucleic acids such as RNA. This aspect also generated an interest in the field of neurodegenerative disorders after the discovery of EVs as mediators delivering microRNAs (miRNAs) in intercellular communication [12] or the source of miRNAs as candidates for biomarkers of disease [13,14].

MiRNAs are endogenous small noncoding RNAs of 21–23nt in length that are capable of controlling gene expression through post-transcriptional regulation. MiRNAs exert their regulatory effect by suppressing translation of mRNA through the binding to the 3′-untranslated region (UTR) of target mRNA or by degrading target mRNA. The same miRNA can target different mRNAs contemporarily, whereas a single mRNA can be regulated by different miRNAs. 

MiRNAs have been identified in many biological fluids, such as plasma, serum, CSF, urine [15], highlighting their potential role as peripheral noninvasive biomarkers of several pathological conditions, including cardiovascular diseases, cancer and neurodegenerative diseases [16,17]. A growing body of evidence demonstrated that they are intimately involved in synaptic function and in specific signals during memory development [15]. Moreover, in vivo experiments showed that Aβ and Tau pathology drove the deregulation of some neuronal miRNAs (miR-142-5p, miR-146a-5p, miR-155-5p), alterations confirmed also in AD patients [18]. MiRNAs could also have a predominant role in driving the pathogenic process of the disease as it was shown that most altered miRNAs also target AD relevant pathogenic proteins [19]. Several miRNAs have been robustly identified as deregulated in brain tissue from AD patients as recently reviewed in Herrera-Espejo et al. [20], whereas others were proposed as circulating peripheral biomarkers of disease, although none of them had the same regulation status in all studies [20]. 

Extracellular miRNAs in serum and plasma are found in different fractions [21]. Usually, they are encapsulated in membrane vesicles or are released from apoptotic bodies [22]. Most circulating miRNAs are, however, bound to proteins such as Argonaute2 [23]. 

The high heterogeneity of the results on circulating miRNAs levels in AD casts a shadow on their real diagnostic potential. Moreover, the origin of circulating miRNAs is heterogeneous itself and likely could not reflect the specific pathological status. Lastly, independent of the localization that could be protein- or vesicle-bound, miRNAs in serum or plasma could hardly trace back their cellular origin.

In this scenario, small NDEVs could be considered a promising source of miRNAs that could directly reflect the physiological condition of the nervous system without introducing confounding factors. 

It was already proven that EVs represented an enriched source of noncoding RNAs of different types, such as miRNAs, that, in this way, result protected from RNase degradation. This peculiar aspect represents the solid foundation for their clinical application as diagnostic biomarkers. Moreover, the process of packaging of miRNAs into small EVs in cytoplasm is a finely regulated event that includes multiple steps, supporting the active functional role of miRNAs in these vesicles.

MiRNAs released from EVs could modulate the expression and function of amyloid precursor protein and tau proteins. EV-carried miRNAs could drive, via Toll-like receptors, inflammatory processes in AD and may also regulate neuroplasticity to relieve neurological damage [24].

Moreover, Lugli et al. found a consistent dysregulation of miRNA levels in plasma-derived EVs from AD patients pointing out a specific signature of seven miRNAs that were able to predict the group identity [12].

Given these premises, herein we carried out a comprehensive characterization of small NDEVs and a further analysis of their miRNA content for the detection of possible dysregulated miRNAs in patients with AD. We evaluated simultaneously miRNAs content from total plasma small EVs and small NDEVs to be certain of specifically isolating those of neuronal derivation from the others deriving from the total small EVs population. 

Our final goal was to detect a specific small NDEVs miRNA signature in plasma from patients with AD. Assuming that pathogenic alterations in such NDEVs may reflect the brain environment, this research could lead to the identification of potential reliable peripheral biomarkers for early diagnosis of the disease. 

## 2. Methods 

### 2.1. Study Design and Sample Collection 

Forty patients with AD were recruited at the Alzheimer Center of the University of Milan, Fondazione Cà Granda, IRCCS Ospedale Maggiore Policlinico, between 2015 and 2017. All patients underwent a clinical interview, neurological and neuropsychological examination, routine blood tests, brain MRI and lumbar puncture (LP) for quantification of the CSF biomarkers Aβ, total tau and tau phosphorylated at position 181 (Ptau). Cut-off thresholds of normality were: Aβ ≥ 600 pg/mL; tau ≤ 500 pg/mL for individuals older than 70 years and ≤450 pg/mL for individuals aged between 50 and 70 years; Ptau ≤ 61 pg/mL [16]. The clinical diagnosis of AD was supported by CSF signature, consisting of decreased Aβ and increased tau and Ptau levels, according to the criteria of the International Working Group [2]. Details of the biomarker levels of all participants are provided in Supplementary Table S1. The Clinical Dementia Rating (CDR), the Mini Mental State Examination (MMSE), the Frontal Assessment Battery (FAB), the Wisconsin Card Sorting Test (WCST), and the Tower of London test assessed cognitive dysfunctions. The presence of significant vascular brain damage was excluded (Hachinski Ischemic Score < 4). Forty plasma samples from sex-matched healthy controls (CTRLs) were also collected. All individuals underwent LP in suspicion of a CNS disease and were discharged with no evidence of neurological diseases and cognitive impairment. None of them developed cognitive decline over time and, at inclusion, MMSE was ≥28. Whole blood samples, collected at the time of diagnosis, were allowed to sit at room temperature for a minimum of 30 min and a max of 2 h, after collection. Separation of the clot was done by centrifugation at 1000–1300× *g* at room temperature for 15–20 min. Plasma was removed and dispensed in aliquots of 550 µL into cryo-tubes and stored at −80 °C until use. Informed consent to participate in this study was given by all subjects or their caregivers. The study was approved by the local ethics committees (Parere 66/2016 Ethics Committee IRCCS Fatebenefratelli and Parere 532_2019bis del 13-6-2019 - Comitato Etico Milano Area 2).

### 2.2. Isolation of Total EVs from Plasma

Aliquots of 500 µL of plasma were centrifuged at 3000× *g* for 15 min to remove cells and debris The supernatant was transferred to a sterile vessel and incubated with 5 µL of purified thrombin ([500 U/mL], System Biosciences) to a final concentration of 5 U/mL for 5 min at room temperature. This step is required to remove the large amount of fibrin present in the plasma. After incubation, samples were centrifuged at 10,000 rpm for 5 min. Supernatants were incubated with 126 µL of ExoQuick precipitation solution (EXOQ; System Biosciences) and refrigerated for 30 min at 4 °C. The resulting suspension was centrifuged at 1500× *g* for 30 min at room temperature. The pellet was then re-suspended in 500 µL of 0.2 µm filtered 1X PBS and conserved at −80 °C until use. 

### 2.3. Selective Capture of CD81 and L1CAM-Positive Small Neuronal-Derived EVs (NDEVs) from Plasma with Magnetic Beads and Sorting of EVs Based on Flow Cytometric (FACS) Analysis

The Exo-Flow Exosome Capture kit (System Biosciences) for the selective capture and flow sorting of NDEVs purification based on a particular surface marker was used according to the instruction of the manufacturer. The procedure enables selective capture for immunopurification and flow sorting to separate the distinct subpopulations of small EVs, based on the L1CAM or CD81 surface marker. Previously isolated small EVs were resuspended and bound to the magnetic beads for selective capture and subsequent FACS analysis and sorting. The specific beads involved have 9.1 µm diameter, a characteristic that enables great vesicle capture. 

Supernatants containing the eluted vesicles were carefully removed and transferred to fresh tubes for further analyses. Supernatants were subjected to sorting by cell sorting (FACSAria SORP and FACSDiva software, Becton Dickinson, San Josè, CA) and L1CAM NDEVs were selected. In particular, small NDEVs were identified based on a gating strategy, including a first region on physical properties and a second one on FITC fluorescence intensity. Specifically, the protocol involves, for each sample and the negative control, the addition of the bead slurry (40 μL) to 1.5 mL tubes and the further incubation on a magnetic stand for 2 min. Then they were washed twice. The tubes with bead slurry were removed from the magnetic stand and 10 μL of mouse anti-human CD171 (L1CAM, neural adhesion protein) biotinylated capture antibody (clone 5G3, eBioscience) and anti-human CD81 (System Biosciences), both at a concentration of 100 ng/µL in 1X PBS, were added. After mixing, the tubes were incubated on ice for 2 h with gentle mixing by flicking every 30 min. The capture antibody-beads were suspended with 400 μL of wash buffer. The plasma isolated EVs (or PBS for negative control) were added to bead samples to a final total volume of 500 μL and placed on a rotating shelf at 4 °C overnight for capture. A portion of 240 μL of exosome stain buffer and 10 μL exo-fluorescein isothiocyanate (FITC) exosome staining solution were added to the beads and incubated on ice for 2 h with gently flicking every 30 min to mix. Samples were washed 3 times in place on the magnetic stand, followed by the addition of 300 μL wash buffer for FACS analysis, without vortexing. Flow-sort stained EV/bead complexes were incubated with 300 μL of exosome elution buffer for 40 min at room temperature on a rotating rack. Finally, the supernatants containing L1CAM positive EVs were obtained. 

### 2.4. Transmission Electron Microscopy (TEM)

A total of 10 μL of EV-enriched solution was placed on a copper mesh and incubated for 10 min at room temperature. The grids were then dried by placing them sideways on filter paper and the EV-enriched fraction was contrasted by using a solution of saturated uranyl acetate in water for 5–10 min. Samples were subsequently dried. The copper mesh was then observed, and pictures captured with a transmission electron microscope (Leo912ab 80 kv).

### 2.5. Characterization of Small NDEVs by Nanoparticle Tracking Analysis (NTA)

Suspensions containing total small EVs and small NDEVs from patients and controls were analyzed using the Nano-Sight NS300 instrument (Malvern, Worcestershire, UK), a laser-based, light-scattering system which provides a reproducible platform for nanoparticle characterization. Prior to analysis, samples were diluted in PBS to obtain a range of 20–200 particles in the field of view (particles per frame, PF). For each sample, 5 videos of 60 s duration were taken. Data were processed using NanoSight NTA Software 3.2 (Malvern, Worcestershire, UK). The NTA post acquisition settings were optimized and kept constant between samples. Data obtained were particle size distribution (D-values; D10, D50 and D90: a particle size value indicating that, respectively, 10%, 50% and 90% of the distribution is below this value) and concentration (particles/mL).

### 2.6. Total Plasma EVs Protein Quantification

The protein concentration of total plasma EVs, extracted from two samples, was quantified by Micro BCA Protein Assay Kit (ThermoFisher Scientific, Waltham, MA, USA) following the manufacturer’s specifications and using BSA (ThermoFisher Scientific, Waltham, MA, USA) as a standard. Values were extrapolated from this curve, using a third-order polynomial equation, with r^2^ > 0.98 for each assay.

### 2.7. Western Blotting

We performed Western blotting analysis according to standard protocols. Briefly, EVs were dissolved in PBS with LDS sample buffer (Life Technologies, Waltham, MA, USA) and separated using the 4%–12% Bolt Bis-Tris Precast Gels (Life Technologies, Waltham, MA, USA) with MOPS SDS running buffer (ThermoFisher Scientific, Waltham, MA, USA). Then samples were electro-transferred onto PVDF membranes (ThermoFisher Scientific, Waltham, MA, USA) for 2 h at 60V at room temperature and the membranes were immunoblotted with primary antibodies overnight and then incubated with horseradish peroxidase-conjugated secondary antibodies (1:10,000; Pierce, ThermoFisher Scientific, Waltham, MA, USA) for 1 h. Immuno-positive bands were detected by enhanced chemiluminescence (Pierce, Thermo-Fisher Scientific) according to the manufacturer’s instructions. The primary antibodies anti-L1CAM (1:1000), anti-TSG101 (1:1000) and anti-VPS35 (1:1000) came from Abcam (Cambridge, MA, USA), and anti-CD9 (1:1000) was purchased from Santa Cruz Biotechnology (Dallas, TEX, USA). 

### 2.8. RNA Extraction from Total and Neural Derived Plasma EVs

Total RNA was extracted by using Total Exosome RNA and Protein Isolation Kit (Thermo Fisher Scientific, Waltham, MA, USA) that uses Acid-Phenol/Chloroform extraction to provide a robust front-end RNA purification step, followed by a final RNA purification over a glass-fiber filter. Ethanol was added to samples that were passed through a Filter Cartridge containing a glass-fiber filter which immobilizes the RNA. The filter was washed, and the RNA eluted with 30 μL low ionic-strength solution according to the manufacturer’s instructions.

The RNA from total small EVs was analyzed using Agilent 2100 Bioanalyzer and RNA 6000 Nano kit (Agilent Technologies, CA, USA) for the determination of RNA concentration, purity, and integrity.

### 2.9. Expression Analysis of microRNA by TaqMan OpenArray Human Advanced MicroRNA Panel

Total RNA was reverse transcribed using the TaqMan Advanced miRNA cDNA Synthesis Kit according to the instructions of the manufacturer (Thermo Fischer Scientific, Waltham, MA, USA). Given the low RNA amounts obtained from NDEVs, cDNA was preamplified prior to the final real-time PCR step. The preamplification step was performed using the TaqMan PreAmp Master Mix and pooled gene-specific primers. The preamplified cDNA (diluted 1:20) was mixed with 2X TaqMan OpenArray Real-Time Master Mix to perform real-time PCR on the OpenArray plate. A 5 μL sample of PCR reactions were distributed into each well of 384-well plate and then samples with the master mix were loaded from the 384-well sample plate onto the OpenArray plate using the OpenArrayAccuFill System. Finally, PCR was run on the QuantStudio 12K Flex Real-Time PCR System (Thermo Fischer Scientific, Waltham, MA, USA). For single-tube expression analysis, specific miRNA Advanced TaqMan probes were used (478532_mir, 477983_mir, 478619_mir, 478358_mir, 478293_mir) according to the instructions of the manufacturer.

### 2.10. RNase Treatment and Protection Assay 

Small EVs isolated from 4 samples (total plasma and NDEVs) were used for miRNA RNase protection assay. Three conditions for the same sample were used prior to RNA extraction. The first condition was EVs treated with 20 mU/mL RNase I in order to eliminate all free circulating RNAs; the second condition consisted of EVs treated with 20 mU/mL RNase I and 1% TritonX-100 to disrupt membranes. The third consisted of EVs without treatments used as controls. All samples were incubated at room temperature for 20 min on a rocking wheel [25,26]. After incubation the RNA was extracted and the miRNA gene expression assay was carried out as previously described [16].

### 2.11. Target Gene Prediction and Bioinformatics Analysis

For miRNAs confirmed in the validation phase, target prediction was performed using TargetScan v 7.1 [27]. DIANA-mirPath was used to perform target prediction and pathway analysis based on two algorithms, microT-CDS and miRTarBase. The software performs an enrichment analysis of multiple miRNA target genes to the Kyoto Encyclopedia of Genes and Genomes (KEGG) pathways. The graphical output of the program provides an overview of the parts of the pathway modulated by selected miRNAs. The statistical significance value associated with the identified biological pathways was calculated by mirPath [28]. Next, the tissue location of enriched expression for targets was determinate using FunRich v.3.0 (www.funrich.org) which combines multiple established databases including UniProt, Human Protein Atlas, Human Proteome Browser, Human Proteome Map, ProteomicsDB and Human Proteinpedia to infer regional and cell-type enrichment of target mRNAs for a given list of miRNAs [29].

### 2.12. Normalization and Statistical Analyses 

Exploratory data analysis was performed using ClustVis (https://biit.cs.ut.ee/clustvis/).

Normalized delta Cq-values (dCq) were used for clustering analysis based on Euclidean distance and complete linkage. Unit variance row scaling was applied for hierarchical clustering and principal component analysis (PCA) [30,31].

Results from the real time PCR will be calculated as Fold-Change [2^(-ΔΔCt)], which is the normalized gene expression [2^(-ΔCt)] in the AD group divided by the normalized gene expression [2^(-ΔCt)] in the control group.

GraphPad Prism 6.0 software (San Diego, CA, USA) was used for statistical analysis. Fisher’s exact test and the student’s t-test were used to compare gender and age distribution. Test of normality was performed by using the Shapiro−Wilk’s test. Subsequently, comparisons of ncRNAs levels, expressed by relative quantification (RQ), between patients and controls were performed by using the nonparametric Mann−Whitney test and the correction for multiple comparisons was performed using the post hoc Dunn’s test. The selection of miRNAs for normalization was performed using the algorithm NormFinder. From this, the spike-in cel-miR-39 as well as miR-20b-5p and miR-21-5p were used to normalize across all Ct values using a combined geometric mean of all three Ct values [32].

### 2.13. Availability of Data and Materials

All data relevant to the study are included in the article or as supplementary information. Upon reasonable request, additional information (e.g., protocols) will be shared by the corresponding author.

### 2.14. Ethics Approval and Consent to Participate

All subjects gave written consent and agreed to be in the study, and biological specimens were obtained after consent with approval of local CE: Parere 66/2016 Ethics Committee IRCCS Fatebenefratelli and Parere 532_2019bis del 13-6-2019 - Comitato Etico Milano Area 2.

## 3. Results

### 3.1. Characterization of Total and L1CAM Positive EVs from Plasma

A population consisting of 40 AD patients and 40 healthy controls (CTRLs) was investigated. The study was structured as follows: (1) discovery phase involving 20 AD patients and 20 CTRLs tested for 754 candidate miRNAs and (2) validation phase, involving the remaining 20 AD samples and 20 controls, for the validation of previously determined best hits miRNAs.

Participants’ demographic and clinical information are summarized in Table 1. Small EVs fractions were characterized for the presence of specific surface markers, size, morphology with three different experiments as suggested by the ISEV [3]. Initially, the size of the plasma derived EVs, including the neural-derived fraction, was evaluated with NTA, then different vesicle markers were tested by Western blotting. Additionally, TEM analysis for total and small neural-derived EVs was performed. 

**Figure 1:**
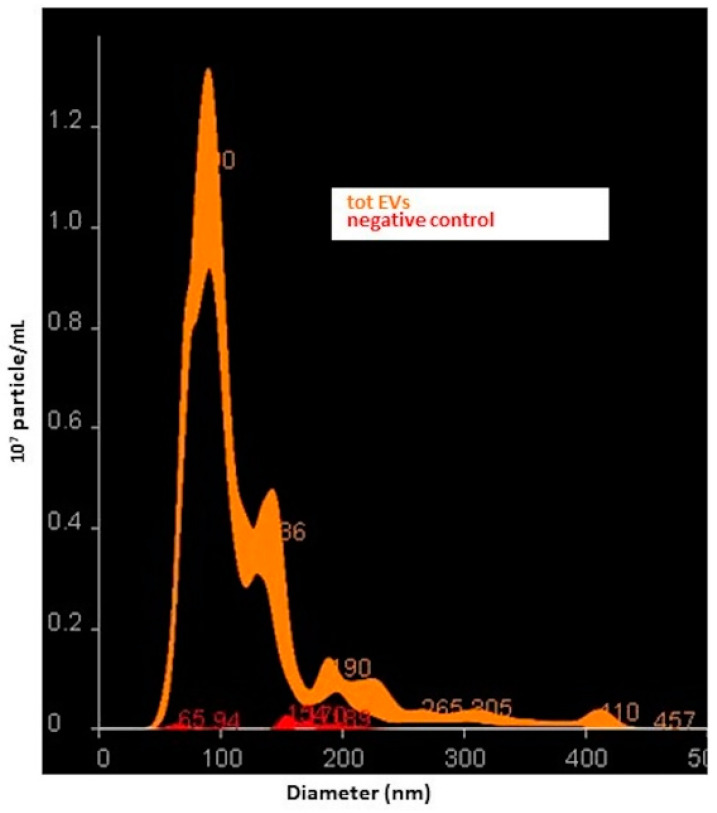
Nanoparticle tracking analysis (NTA). Representative outcomes of total small extracellular vesicles (EVs) suspension and its negative control are reported.

NTA confirmed that the total EV suspensions were enriched in small EVs. In particular, NTA revealed the following size distribution (mean ± SD): D10: 70 ± 1.3 nm; D50: 97 ± 1.9 nm; D90: 196 ± 9.9 nm with a particle concentration of 6.7 ± 0.8 × 10^11^ particles/mL); the negative control was undetectable, i.e., below the detection limit of the instrument, thus not interfering with the sample analysis (Figure 1). The protein concentration of the total plasma EVs was also evaluated in two samples, and an EV-associated protein concentration of 189.67 ± 98.2 μg/mL was reported. The ratio of particle counts and protein concentration as described by Webber and Clayton, 2013, was calculated, in order to test the quality of the purification process [33]. A ratio of 3.5 × 10^9^ p/μg was obtained suggesting good purity of the total plasma EV preparations [33]. 

A substantial dimensional heterogeneity was detected by isolation with Exoquick, attributable to different types of vesicles, including exosomes, classified as under 100–150 nm, and larger microvesicles, usually above 100–150 nm. Due to the difference in the detected vesicle size and according to the “minimal information for studies of EVs (MISEV)” guidelines it was chosen to refer to them as small EVs [3].

NTA analysis on small NDEVs was not feasible, since the negative control had a signal intensity overlapping with the one from NDEVs suspension (Supplementary Figure S1) possibly due to antibody aggregates’ cross-reactivity.

**Figure 2:**
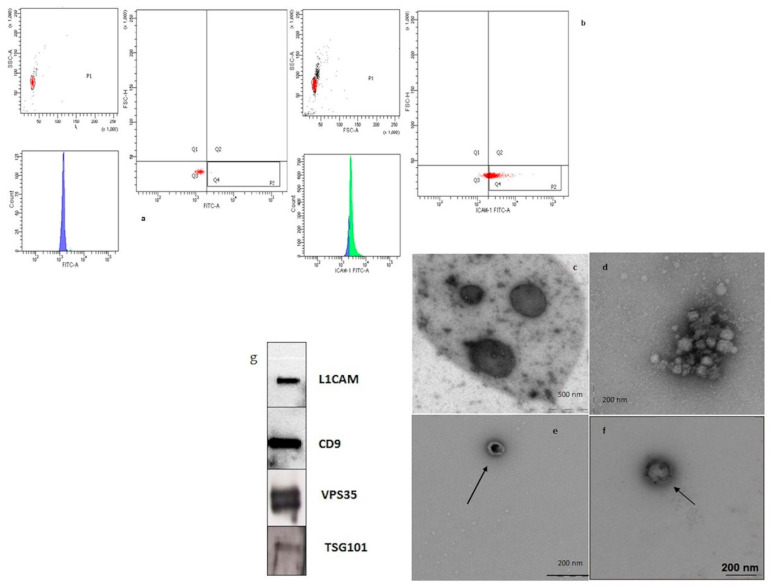
Total and neural-derived extracellular vesicles (NDEVs) enriched fractions from plasma. A and B. Flow cytometry analysis dot-plot. Panel (**a**) represents the negative control, (**b**) the neural-derived EVs from plasma of one AD patient. The EVs are stained with the fluorophore FITC. In Panel (**a**) there is an absence of fluorescent emission FITC (green peak), which instead is present in the positive sample panel (**b**). The same result is shown in the dot plot. Indeed, in the negative control the bead-Ab system is located in the quadrant Q3, in the absence of FITC fluorescence, while in the sample the signal shifts in the Q4 quadrant, where the FITC fluorescence is positive. C, D, E, F TEM analysis. Ultrastructural examination of fractions enriched in EVs (**c**) CD81-positive, (**d**) of neural derivation (L1CAM positive). TEM images showed that EVs were oval or oval or bowl-shaped capsuled. No substantial difference in the morphology of EVs isolated from fresh samples (**e**) compared with frozen ones (**f**). Western blot analysis. L1CAM, CD9, VPS35, TSG101 markers were all detected in the NDEVs enriched fraction of plasma samples (**g**).

The neural-derived enriched fraction was isolated from the plasma of all the AD patients and all the healthy controls by using the neuronal marker L1CAM after sorting by flow cytometry (Figure 2a,b). The same approach was used to isolate total EVs present in plasma that were selected for the general marker CD81. In order to confirm the presence and the origin of the isolated vesicles, NDEVs and total EVs underwent further steps of characterization. 

TEM revealed a homogenous population of morphologically distinctive particles, mainly oval-shaped, approximately 150 nm in diameter of EVs labeled with anti-CD81, known as general small vesicle marker, and L1CAM marker (Figure 2c,d). Furthermore, we did not find substantial differences in the morphology of EVs isolated from fresh samples (e) compared with frozen ones (f). Western blot analysis confirmed the presence of the neural marker L1CAM in the extracted vesicle subpopulation. As shown in Figure 2g, we found NDEVs immunoreactivity for both L1CAM and the common vesicle markers CD9, Tsg101 and the protein retromer Vps35.

### 3.2. MiRNAs Expression Profile in Total Plasma EVs and NDEVs in AD Patients and Healthy Subjects

**Figure 3:**
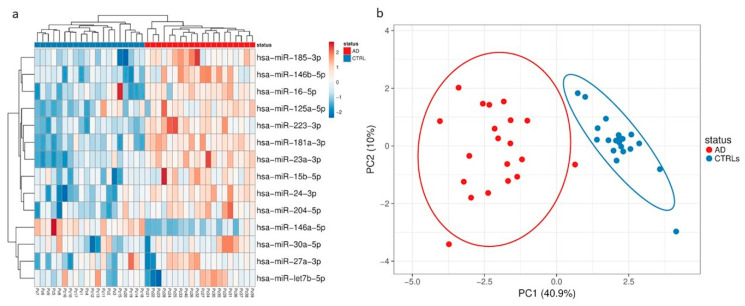
(**a**) Hierarchical clustering of samples based on microRNA (miRNA) profiles observed in total plasma EVs from Alzheimer’s disease (AD) patients and control subjects (CTRLs). The most variant (according to the coefficient of variation) miRNAs were used for clustering analysis (Pearson correlation, average linkage). Rows are centered and unit variance scaling is applied to normalized expression values. Color indicates relative up- (red) or downregulation for each miRNA (row). (**b**) Principle component analysis based on the most variant miRNAs in total plasma EVs.

**Figure 4:**
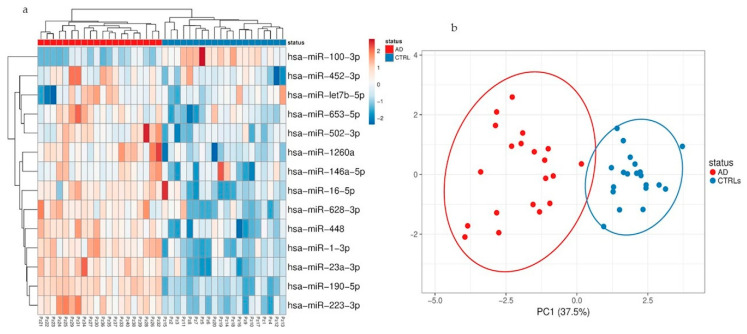
(**a**) Hierarchical clustering of samples based on microRNA (miRNA) profiles observed in total plasma EVs from AD patients and CTRLs. The most variant (according to coefficient of variation) miRNAs detected in NDEVs were used for clustering analysis (Pearson correlation, average linkage). Rows are centered and unit variance scaling is applied to normalized expression values. Color indicates relative up- (red) or downregulation for each miRNA (row). (**b**) Principle component analysis based on the most variant miRNAs in NDEVs.

RNA from total plasma EVs and NDEVs was initially isolated from 20 AD samples and 20 controls and the quality and size of extracted RNA were verified by bioanalyzer (Supplementary Figure S2). Thereafter, miRNAs were profiled by RT-qPCR using the TaqMan Open Array miRNA panel, which enables the testing of 754 human miRNAs. Raw data underwent quality control measures. Applying a Crt cutoff < 28 and the amp score > 1 (measures of good amplification quality), a total of 57 miRNAs were detected across all 40 samples (Supplementary Table S2). For the normalization, Crt values of spike-in cel-miR-39 and miR-20b-5p along with the endogenous miR-21-5p were combined to generate the geometric mean using the available protocol of Andersen [32]. A specific distribution of miRNA expression was observed for both the EV populations analyzed, although there was an overlap for some miRNA expression between the fractions. The most variable miRNAs in the total EVs (Figure 3) and NDEV (Figure 4) enriched fraction were selected based on the coefficient of variation and used for principal component analysis (PCA) and hierarchical clustering. These analyses of all identified miRNAs in both EV fractions revealed that the primary miRNA expression data were influenced by the disease. In particular, PCA showed a separation of samples based on disease status.

**Figure 5:**
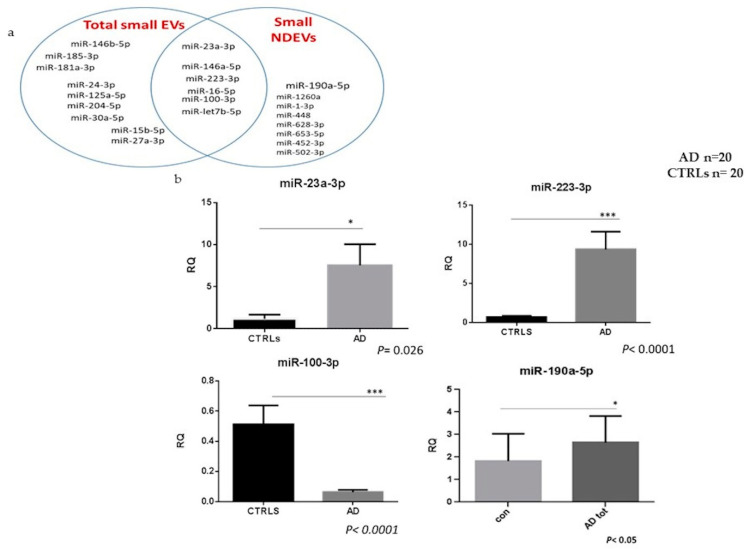
(**a**) The Venn diagram shows the distribution of the most dysregulated miRNAs (> or < 2.5 -fold compared to controls) detected in both subpopulations of EVs. (**b**) Relative quantification of statistically deregulated miRNAs in AD patients (AD *n* = 20 and CTRLs *n* = 20; miR-23a-3p * *p* = 0.026; miR-223-3p *** *p* < 0.0001; miR-100-3p *** *p* < 0.0001) as compared with CTRLs.

As shown in Figure 5a, 59% of miRNAs expressed was found in total plasma EVs, but not in NDEVs, 15% overlapped between the two subpopulations and 26% was found only in the NDEVs (Supplementary Table S2). Specifically, considering the total EVs fraction, increased relative expression levels (greater than 2.5 fold) of miR-146b-5p, miR-181a-3p, miR-24-3p, miR-125a-5p, let-7b-5p and miR-27a-5p, miR-185-3p were observed (Figure 3b) in patients versus CTRLs, whereas decreased relative expression levels (lower than 2.5 fold) of miR-16-5p, miR-15b-5p, miR-30a-5p and miR-204-5p were detected, although the statistical significance was not reached. 

Considering miRNAs detected in NDEVs, a specific signature was observed: miR-1260a, miR-1-3p, miR-448, miR-628-3p, miR-653-5p, miR-452-3p, miR-502-3p and miR-190a-5p were in fact specifically detected in the neuronal-derived EVs fraction and an overall upregulation (greater than 2.5 fold) was observed in patients compared to CTRLs. Moreover, a Mann−Whitney test uncovered a statistically significant upregulation of miR-23a-3p, miR-223-3p, miR-190-5p (1.16 ± 0.49 versus 7.54 ± 2.5, *p* = 0.026; 9.32 ± 2.27 versus 0.66 ± 0.18, *p* < 0.0001; 2.9 ± 1.2 versus 1.93 ± 0.9, *p* < 0.05, respectively) comparing AD patients with CTRLs whereas a statistically significant downregulation of miR-100-3p in AD patients was observed in the same group of EVs (0.069 ± 0.01 versus 0.5 ± 0.1 *p* < 0.0001, Figure 5b). 

In order to confirm the specific vesicle-packed origin of these miRNAs, an RNase protection assay was performed prior to RNA extraction on the specific miRNAs, miR-223-3p and miR-190a-5p, miR-23a-3p, previously detected.

**Figure 6:**
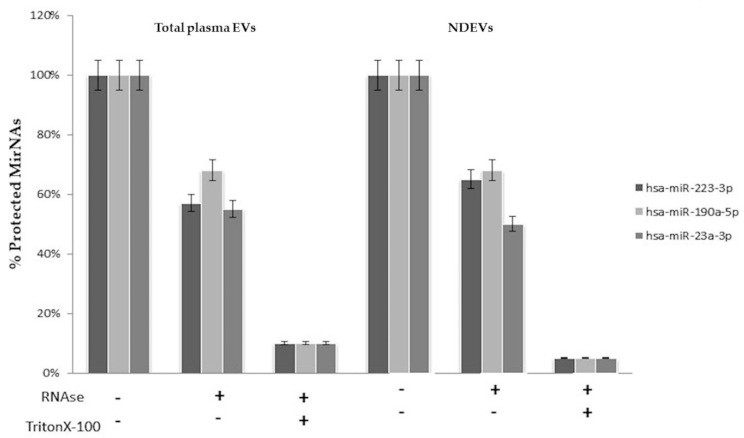
RNase protection assay. The miR-223-3p, miR-190-5p and miR-23a-3p were quantified by q-PCR in total and neural-derived plasma EVs. EVs were treated with or without RNase I and/or Triton X-100 (*n* = 4).

Results showed that the miRNAs expression level was slightly decreased by treatment with *RNase I*, but it was mostly decreased when detergent was added to disrupt the membranes. These results suggested that miRNAs were all well protected by the EV membrane in total plasma as well as the neuronal-derived ones (Figure 6). These data revealed that a significant amount of miR-223-3p, miR-190a-5p and miR-23a-3p existed in a vesicle associated manner.

To deepen the effective robustness of statistically significant NDEVs’ miRNA expression results (shown in Figure 5b), a further gene expression analysis of miR-23a-3p, miR-223-3p, miR-190-5p and miR-100-3p was done on NDEVs extracted from the validation cohort samples consisting of 20 AD patients and 20 CTRLs. 

**Figure 7:**
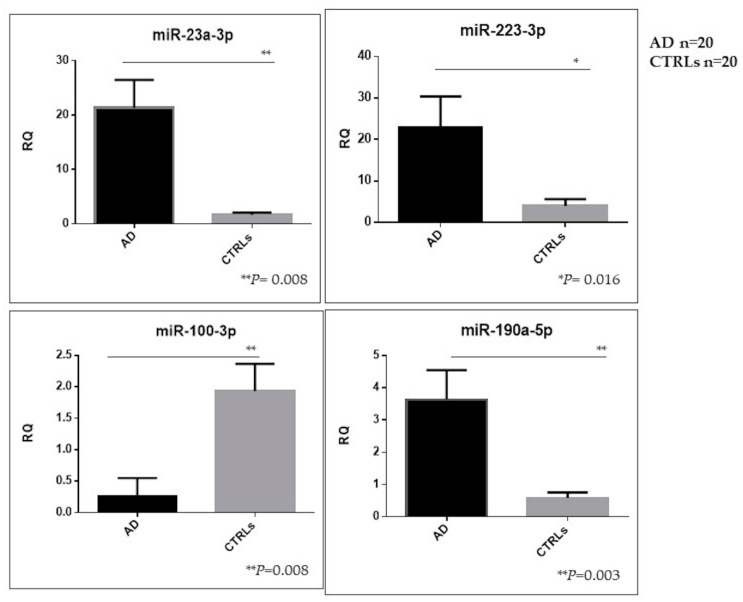
Relative expression of best dysregulated miRNAs tested in NDEVs from the remaining 20 AD patients and 20 CTRLs (miR-23a-3p ** *p* = 0.008; miR223-3 * *p* = 0.016; miR-190a-5p ** *p* = 0.003; miR-100-3p ** *p* = 0.008).

Significant upregulations of miR-23a-3p, miR-223-3p and miR-190a-5p levels in NDEV from AD patients as compared with controls were confirmed (*p* = 0.008; *p* = 0.016; *p* = 0.003, respectively) as well as significant downregulation of miR-100-3p levels (*p* = 0.008) (Figure 7). 

### 3.3. miRNA Targets and Pathways Prediction

**Figure 8:**
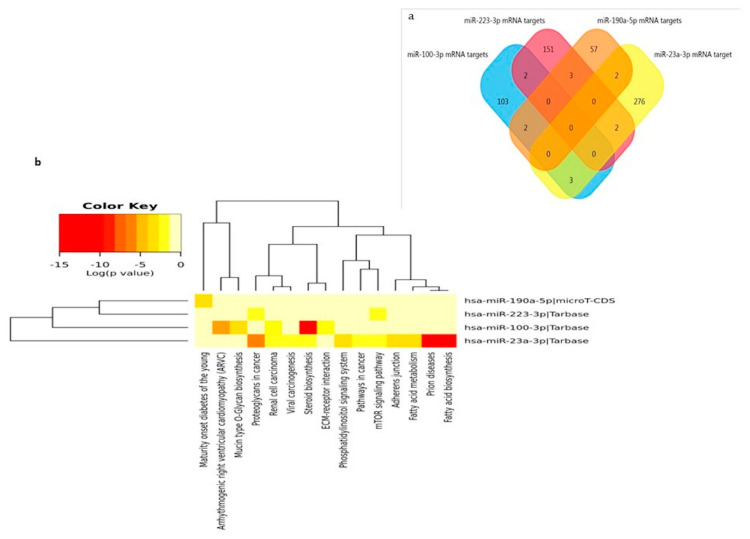
The miRNA targets and pathways prediction. (**a**) A 3-set Venn diagram overlap of mRNA targets statistically deregulated in NDEVs in AD patients (miR-23a-3p, miR-223-3p, miR-100-3p. and miR-190a-5p. (**b**) Heatmaps of significant pathways for statistically significant NDEV miRNAs, predicted by DIANA-miRPath (v.3.0). Pathways are depicted on the *x*-axis and miRNAs on the *y*-axis. The color code represents the log (*p*-value), with the most significant predicted miRNA-pathway interactions in red.

The differentially deregulated microRNAs in patients were screened for their possible target genes and biological pathways using the TargetScan v7.1 algorithm. According to the analysis, a total of 1332 mRNA targets for miR-23a-3p, 412 for miR-223-3p, 3168 for miR-100-3p and 224 mRNA targets for miR-190a-5p were identified. Overlaps between mRNA targets is evident between the combination of two miRNAs, but no targets are shared among all of them (Figure 8a). 

Analysis with DIANA-miRpath (v.3.0) showed that combinations of at least two miRNAs overlapped in pathways such as steroid biosynthesis, proteoglycan in cancers, mTOR signaling and prion disease pathway (Figure 8b). 

**Figure 9:**
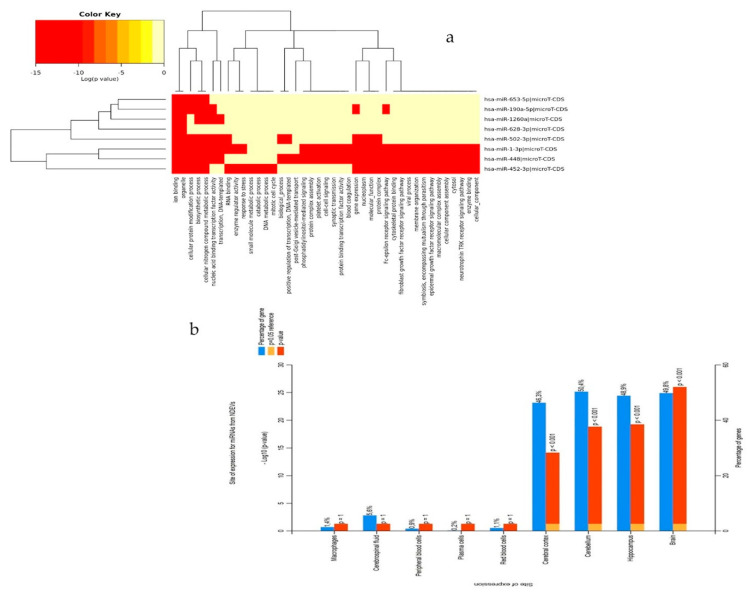
(**a**) Heatmaps of significant pathways predicted by DIANA-miRPath (v.3.0) for specific miRNAs detected in NDEVs. Pathways are depicted on the *x*-axis and miRNAs on the *y*-axis. The color code represents the log (*p*-value), with the most significant predicted miRNA-pathway interactions in red. (**b**) Tissue site of expression determined using FunRich v3.0 (http://funrich.org/). The percentage of targets showing regional enrichment (blue bar) and the significance of regional enrichment (red bar). Top *x*-axis is –log10 (*p*-value) of regional enrichment; bottom *x*-axis is the percentage of targets overlapping with the region.

For miRNAs specifically detected in NDEVs, the analysis with DIANA-miRpath identified overlapping specific CNS pathways considering at least three miRNAs, such as the glioma, axon guidance, long term depression and calcium signaling ones (Figure 9a). To assess brain region and cell-type specific localization of NDEVs miRNAs, the FunRich v.3.0 online available tool was used [29]. Results revealed that mRNAs targets were depleted in peripheral blood cells, macrophages and red blood cells, as expected. Notably, the markers of the brain, hippocampus, cortex and cerebellum were instead enriched (Figure 9b)

## 4. Discussion

Herein, we performed for the first time a comprehensive characterization of small NDEVs, and identified a specific AD signature, consisting of significantly increased levels of miR-23a-3p, miR-223-3p, miR-190a-5p and decreased levels of miR-100-3p. 

The need for peripheral biomarkers derives from the invasiveness of the procedure for CSF collection. In this scenario, NDEVs are candidates for harboring biomarkers reflecting CNS pathogenic AD alterations. 

Recent studies showed that the NDEV enriched fraction represents a powerful reservoir of biomarkers for the investigation of key molecules involved in the pathogenesis of AD and related dementias [7,8,9]. 

The methodology developed by Fiandaca et al. [7] for the isolation of small NDEVs represents a modification of the original one published by Mitsuhashi, et al. [34], pioneers in the detection of exosomes in plasma from specific cellular derivation. The protocol basically involves an immunochemical enrichment of exosomes from a neural source using cocktails of biotinylated antibodies and subsequent steps of incubation with streptavidin agarose resin. Despite the published protocol being successfully applied for the determination of different proteins in neuronal-derived exosomes, there is still a lack of standardization of the method. Conversely, the methodology herein described could be easily approached for the harmonization of a shared protocol among laboratories, as it is based on the use of commercially available products already proven to isolate exosomes with a good reliability and to be adaptable to the needs of specific enrichment for a neuronal population of extracellular vesicles [35]. To provide a complete and objective assessment of this methodology, it is important to mention that this isolation protocol has an important impact on the quality of isolated EVs [36], and on the amount of miRNAs obtained from the EVs’ preparation [37].

However, since NTA analysis revealed a significant dimensional heterogeneity among the detected vesicles, we chose to address them as small EVs as suggested by the MISEV guidelines [3].

Regarding the current data available in literature on specific miRNAs as biomarkers of neurodegenerative diseases, there is limited evidence of an altered expression of plasma miRNAs in EVs from AD patients [12,24].

Recently, Chat et al. found miR-212 and miR-132 significantly downregulated in small NDEVs from AD patients using a relatively small sized SYBR green array based analysis testing 372 miRNA candidates [38].

However, a comprehensive high throughput miRNA investigation considering either small NDEVs and the plasma total ones has not been performed so far.

In the current study, for the first time, miRNA content associated to the specific NDEV population was analyzed, and we showed that miRNA NDEVs’ cargo is different from that deriving from the overall EV population. Therefore, we identified a specific neuronal signature in peripheral circulation, likely associated to brain miRNA pathological dysregulations. As expected, due to the low amount of the specific neural-derived extracellular population and the subsequent relative RNA, only 26% of miRNAs investigated was reliably detected. All the miRNAs detected were already found in the extracellular vesicles from human biological samples [39,40]. In particular, miRNAs specifically found in NDEVs were already reported to be expressed in exosomes isolated from CSF [41]. 

Despite the finding of several dysregulations, both considering the total amount of vesicles and the neural-derived fraction, few miRNAs reached the statistical significance threshold. Among those, miR-23a-3p, miR-223-3p, miR-100-3p and miR-190-5p showed instead a significant dysregulation in NDEVs from AD patients compared with controls. 

Analysis of pathway predictions using bioinformatics tools failed to reveal pathways robustly disease-correlated common to the four miRNAs, although they were demonstrated to be singularly involved in specific processes of the CNS such as axon guidance and long-term depression as well as in neurodegenerative diseases such as AD. A deeper analysis of available literature revealed a long history of correlation among the investigated miRNAs with dementia. MiR-223 for instance was already proposed as one of the candidate biomarkers for AD as free circulating as well as contained in serum exosomes [12,14,42]. In particular, we previously found a significant downregulation of free-circulating miR-223 and miR-23a levels in serum from AD patients [16]; on the other hand, in the present study, increased levels of NDEVs miR-223-3p and miR-23a-3p were found in patients compared with controls. An explanation of these apparently controversial findings could reside in the suggested function of exosomes as mediators of an active intercellular crosstalk. It is known that exosomes are able to load miRNAs selectively by a specific process, thus the EVs-derived miRNA profile is prespecified and not random, and this is related to the fact that EVs induce changes that are preprogrammed in the host cells [43,44,45]. In this case, increased levels of miR-223-3p in NDEVs could represent an effort by neurons to cope with inflammatory events occurring in AD since miR-223-3p is actively involved in immune response as a negative regulator of inflammation [14].

The same significant trend was found for miR-23a-3p levels in NDEVs; in this case results are concordant with its upregulated levels found in brain tissue from AD patients, although a reduction was found, such as free-circulating in serum or in CSF [14,41]. MiR-100-3p levels were found to be downregulated in NDEVs in patients. Evidence in a murine AD model suggests that the role of miR-100 in Aβ related AD pathogenesis may be related to the “ER stress-miRNAs-mTOR” axis [46]. On the contrary, no association was reported so far between miR-190a-5p and dementia. However, it was already detected in the CSF as free-circulating and, regarding its function, a possible modulation of adult neurogenesis and in vivo, contextual memory was proposed [47]. Interestingly, miR-190 was involved in neuroinflammation as its upregulation inhibited the expression of inducible nitric oxide synthase and other proinflammatory cytokines as IL-6, while it increased the expression of anti-inflammatory cytokines such as IL-10 in a Parkinson’s disease mouse model. Furthermore, miR-190-5p reduced neuronal damage and favored the reduction of neuroinflammation in a PD model suggesting an active role in the disease [48].

As neuroinflammation is a common feature of neurodegenerative disease, miR-190 could act as a common dementia marker so its exclusive role in AD as biomarker needs to be further clarified. MiRNAs expression analysis performed with a human miRNA tissue atlas and Funrich v.3.0 revealed some specific miRNA enrichment in specific brain regions such as the hippocampus, cortex and cerebellum and depleted in peripheral blood cells, macrophages and red blood cells, highlighting the relevant role of these miRNAs in brain functions. This miRNA brain-specificity has been nicely reviewed recently by Herrera-Espejo et al. [20] who highlighted specific deregulation in brain tissue of seven miRNAs in AD patients. Interestingly, these miRNAs are involved in the regulation of key pathways, such as axon guidance, longevity, insulin and the MAPK signaling pathway. 

Similarly, also miR-223-3p dysregulation seems to be involved in AD pathogenesis by modulating many other targets involved in differentiation/proliferation (i.e., NFI-A;/EBPbeta, Mef2c) and NF-kB pathways (i.e., STAT3, IKK). Moreover, it was found to contribute to other CNS diseases such as Multiple Sclerosis and PD [49,50].

Conversely, miR-23a-3p was found to be synaptically located in adult rat in vivo studies and is a negative regulator of transcription [51].

Three out of four significantly dysregulated miRNAs had already been detected in the EVs plasma population, but did not reach the statistical threshold, whereas miR-190a-5p was only detected in the neural-derived fraction, thus suggesting that miRNAs from plasma EVs and miRNAs from plasma NDEVs could be considered two different biomarker entities.

In conclusion, the four significant dysregulated miRNAs found in plasma NDEVs were already detected in the CSF and have some functions or may act in pathways related to the CNS, supporting the hypothesis of the usefulness to consider these EVs obtained from blood as a source of biomarkers for CNS pathologies. Given implications with neuroinflammation, it would be worth studying EVs from microglial origin. Nevertheless, at present there are no reliable extracellular vesicle markers for microglia [11]. 

Despite these promising results, some methodological limitations need to be acknowledged. 

In particular, the detection of NDEVs is hampered by the small numbers of brain-derived EVs that are secreted by potentially disease-relevant cells and transported into peripheral blood. These difficulties are derived from the complexity and technical limitations of current EV isolation methods that could influence the yield of RNA, also demonstrated by the low numbers of miRNAs detected.

The above mentioned technical limitations could explain the discrepancies between our results and the ones shown in the recent paper from Cha et al., [38]. The methodologic procedures, although considering the same L1CAM marker for selecting small NDEVs, were quite different as well as the criteria for patient selection itself that could represent a limitation as underlined by the author. Moreover, the authors highlighted the impossibility of completely ruling out the presence of some plasma RNA outside the vesicles as they did not perform any RNAse protection assay.

All these aspects underline the importance of a standardized procedure and the need for consensus guidelines for the analysis of RNA derived from specific populations of EVs.

Regarding the population considered for this study, there was a statistically different mean age at blood sampling between groups. Nevertheless, this cohort derives from a thorough analysis of all cases who underwent LP in suspicion of neurological disorders and were discharged without any disease. They were followed up over time (mean 3 years) and no cognitive impairment had occurred by follow-up, thus likely excluding the development of memory dysfunctions. Given all these considerations, a further replication step in a larger independent population is required to confirm this approach as a specific “window” on the brain. After that, maybe, other miRNAs or non-coding RNAs molecules could emerge and finally provide a molecular signature with a concrete diagnostic potential. 

## 5. Conclusions

This is the first study that carries out the comparison between a total plasma EVs population and NDEVs in patients with AD, demonstrating the presence of a specific NDEV miRNA signature. In particular, significantly increased levels of miR-23a-3p, miR-223-3p, miR-190a-5p and significantly decreased miR-100-3p levels were observed in NDEVs isolated from AD patients, suggesting a possible “new peripheral window”, possibly able to reflect pathogenic alterations occurring in the brain.

## Figures and Tables

**Table 1:** Characteristics of patients and controls.

	AD	CTRLs
Number of subjects	40	40
Gender (M:F)	15:25 *	22:18 *
Mean age at blood	72.8	66.6
sampling (years ±SD)	2.8 **	5.1 **
Mean Aβ-42 (pg/mL)	510.56	804.98
Mean h-tau (pg/mL)	648.97	548.22
Mean *p*-tau (pg/mL)	86	74

SD = Standard Deviation. * *p* = 0.065; ** *p* < 0.001.

## Supplementary Materials

The following are available online at https://www.mdpi.com/2073-4409/9/6/1443/s1; Supplementary Table S1: table with age distribution and known biomarkers for all participants. Supplementary Table S2: list of miRNAs detected in both subpopulations of EVs by using TaqMan OpenArray^®^ miRNA panel. Supplementary Figure S1: representative outcome of NTA on small NDEVs suspension and its negative control is reported; Supplementary Figure S2: representative Agilent 2100 Bioanalyzer results of EV derived RNA.
