# Supplementary material for: MiRNA Profiling in Plasma Neural-Derived Small Extracellular Vesicles from Patients with Alzheimer’s Disease
Source: Cells. 2020 Jun 10;9(6):1443. doi: 10.3390/cells9061443 (PMC7349735; doi:10.3390/cells9061443)

Supplementary Figure 1: NTA analysis of NDEVs Representative outcome of NTA on neuronal derived small EVs suspension and its negative control is reported.

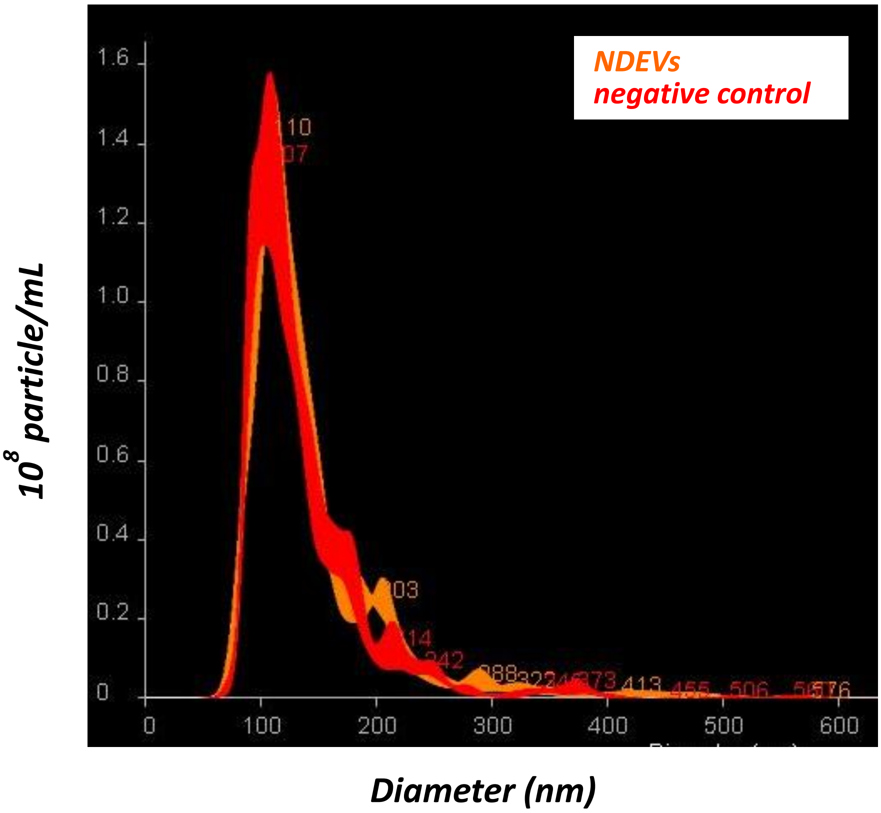

Supplementary Figure 2: Representative Agilent 2100 Bioanalyzer results of EV derived RNA.

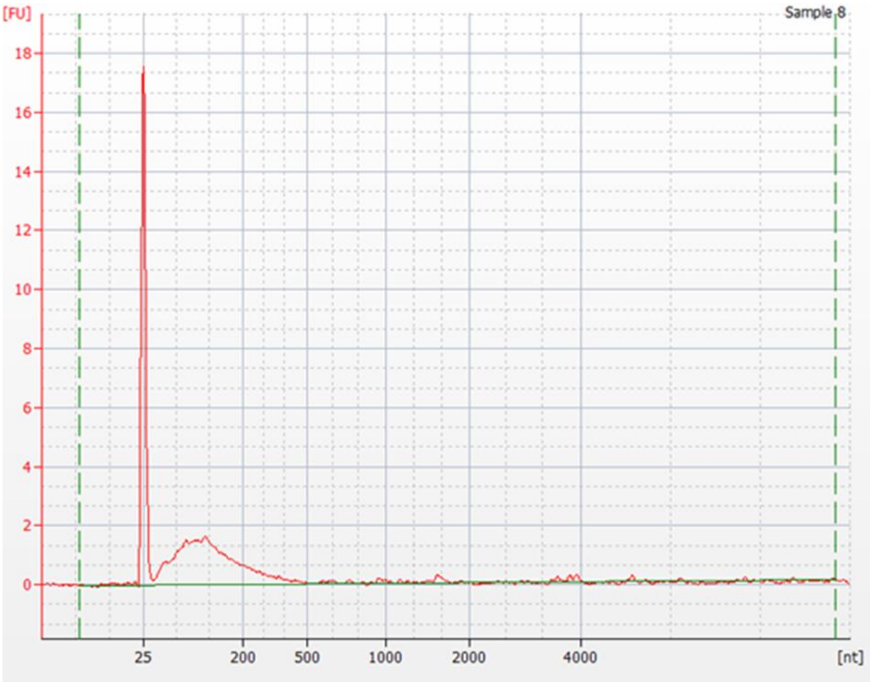

Supplement: Supplementary file 1 [file cells-09-01443-s001.zip › SupplementaryFigures.pdf]
